# Dry Season Transpiration and Soil Water Dynamics in the Central Amazon

**DOI:** 10.3389/fpls.2022.825097

**Published:** 2022-03-24

**Authors:** Gustavo C. Spanner, Bruno O. Gimenez, Cynthia L. Wright, Valdiek Silva Menezes, Brent D. Newman, Adam D. Collins, Kolby J. Jardine, Robinson I. Negrón-Juárez, Adriano José Nogueira Lima, Jardel Ramos Rodrigues, Jeffrey Q. Chambers, Niro Higuchi, Jeffrey M. Warren

**Affiliations:** ^1^National Institute of Amazonian Research (INPA), Manaus, Brazil; ^2^Smithsonian Tropical Research Institute (STRI), Panama City, Panama; ^3^Oak Ridge National Laboratory, Environmental Sciences Division and Climate Change Science Institute, Oak Ridge, TN, United States; ^4^Los Alamos National Laboratory, Los Alamos, NM, United States; ^5^Lawrence Berkeley National Laboratory, Berkeley, CA, United States; ^6^Department of Geography, University of California, Berkeley, Berkeley, CA, United States

**Keywords:** allometry, tropical forests, ecohydrology, root water uptake, basal area, root distribution, sap flow

## Abstract

With current observations and future projections of more intense and frequent droughts in the tropics, understanding the impact that extensive dry periods may have on tree and ecosystem-level transpiration and concurrent carbon uptake has become increasingly important. Here, we investigate paired soil and tree water extraction dynamics in an old-growth upland forest in central Amazonia during the 2018 dry season. Tree water use was assessed via radial patterns of sap flow in eight dominant canopy trees, each a different species with a range in diameter, height, and wood density. Paired multi-sensor soil moisture probes used to quantify volumetric water content dynamics and soil water extraction within the upper 100 cm were installed adjacent to six of those trees. To link depth-specific water extraction patterns to root distribution, fine root biomass was assessed through the soil profile to 235 cm. To scale tree water use to the plot level (stand transpiration), basal area was measured for all trees within a 5 m radius around each soil moisture probe. The sensitivity of tree transpiration to reduced precipitation varied by tree, with some increasing and some decreasing in water use during the dry period. Tree-level water use scaled with sapwood area, from 11 to 190 L per day. Stand level water use, based on multiple plots encompassing sap flow and adjacent trees, varied from ∼1.7 to 3.3 mm per day, increasing linearly with plot basal area. Soil water extraction was dependent on root biomass, which was dense at the surface (i.e., 45% in the upper 5 cm) and declined dramatically with depth. As the dry season progressed and the upper soil dried, soil water extraction shifted to deeper levels and model projections suggest that much of the water used during the month-long dry-down could be extracted from the upper 2–3 m. Results indicate variation in rates of soil water extraction across the research area and, temporally, through the soil profile. These results provide key information on whole-tree contributions to transpiration by canopy trees as water availability changes. In addition, information on simultaneous stand level dynamics of soil water extraction that can inform mechanistic models that project tropical forest response to drought.

## Introduction

The response of tropical forest transpiration (*T*) to changes in environmental conditions remains highly uncertain in Earth System Models due to an unresolved understanding of both abiotic and biotic factors and their interactions (e.g., Berg and Sheffield, 2019). Uncertainty is amplified by the high diversity of tropical tree species (Cardoso et al., 2017) and their differential responses to drought conditions (Cox et al., 2004; Baker et al., 2008; Malhi et al., 2009). Within tropical forests, ecosystem *T* is mediated by tree size and other traits, including stomatal sensitivity to leaf water loss driven by vapor pressure deficit (VPD) (Fontes et al., 2018; Barros et al., 2019; Gimenez et al., 2019; Grossiord et al., 2019). In the Amazon rainforest, studies have reported both drought-induced increases (da Rocha et al., 2009; Brum et al., 2018) and decreases (da Rocha et al., 2009; Fontes et al., 2018) in the rate of sap flow and *T*. While we know that forest water use, i.e., surface evaporation and tree transpiration (evapotranspiration), is influenced by dominant abiotic factors including net radiation, boundary layer conductance, and VPD (Negrón-Juárez et al., 2007; Costa et al., 2010; Brum et al., 2018; Grossiord et al., 2019), it is plant-available water which may explain why evapotranspiration is limited in some regions of the Amazon, but not in others (Fisher et al., 2008; Grossiord et al., 2019).

In the central Amazon, where rainfall is high and the dry season is usually less than 4 months, evapotranspiration generally follows climatic conditions (da Rocha et al., 2009), with rates similar in the dry and wet season (Negrón-Juárez et al., 2007), but can become limited during extensive drought conditions (Fisher et al., 2008). Moreover, given the low soil water holding capacity of the Oxisols that are common in central Amazon, only a short sequence of days without precipitation can lead to the depletion of near-surface soil water. As a result, a greater depth of soil has to be exploited to maintain root water uptake (Hodnett et al., 1995). In the central Amazon, most root distribution is in the upper 20 cm (Ferreira et al., 2002; Noguchi et al., 2014). Yet, water absorption has been exhibited to at least 3.6 m (Hodnett et al., 1995, 1996). In addition, there is evidence of rooting from depths of 6–10 m (Chauvel et al., 1992; Negrón-Juárez et al., 2020). In fact, further east in Pará state, Brazil, tree roots have been found to depths of 18 m (Nepstad et al., 1994).

Differences in access to and use of water are often dependent on tree height and diameter (West et al., 1997; Meinzer et al., 2005). Diameter can be used to predict characteristics related to hydraulic capacity, such as sapwood area (Aparecido et al., 2019), which can then be used to estimate whole tree water use if sap velocity is known. In the tropics, the largest, emergent trees are subject to greater evaporative demand than other shorter canopy trees (Motzer et al., 2005; Kunert et al., 2017) and are a major source of stand-level *T* (Brum et al., 2018). Yet, for canopy dominant and emergent trees, to maintain whole tree water use (*Q*) and photosynthesis during drought requires that they leverage appropriate hydraulic strategies (Esteban et al., 2021; Garcia et al., 2021). Amongst these, root access to deeper water sources is one trait that the largest trees display (da Rocha et al., 2004; Lee et al., 2005; Oliveira et al., 2005; Bruno et al., 2006), although some smaller trees have also been shown to access deeper water sources (e.g., Stahl et al., 2013).

Other traits also influence sap velocity and transpiration dynamics, including relative hydraulic conductivity through the xylem pathway (Fisher et al., 2006), stomatal sensitivity to internal water availability, and external driving forces (Meinzer, 2002; Gimenez et al., 2019). Under drying conditions, tropical trees display a variety of hydraulic responses including avoidance (e.g., leaf drop, or reducing water use by closing stomata—more conservative strategy) and resistance (e.g., maintaining water use at the risk of inducing xylem embolism and hydraulic failure—more acquisitive strategy). Either strategy may ultimately contribute to mortality under excessive drought due to either carbon starvation or hydraulic failure (Rowland et al., 2015; McDowell et al., 2018; Aleixo et al., 2019). Mortality rates vary across the Amazon and are linked to growth rates, physiological failure, and storm damage, which all have some dependence on individual species characteristics (Negrón-Juárez et al., 2017; Esquivel-Muelbert et al., 2020). Understanding variation in *Q* and its response to drought therefore lends insight into relative hydraulic strategies and how they may manifest at the stand level. Knowledge of functional traits and how those traits scale with size can thus provide a pathway for process-level understanding of the controls regulating ecosystem dynamics, including *Q* (e.g., Kotowska et al., 2021). Such trait data and its relationship to stand level evapotranspiration can be highly useful for forest and ecosystem modeling efforts (e.g., Scheiter et al., 2013; Christoffersen et al., 2016).

### Objectives

The objective of this work is to better understand the seasonal patterns of tree water use as linked to upper soil water availability during a dry period in the upland soils of the central Amazon. Our specific questions are as follows: (1) Do different co-occurring canopy species increase or decrease water use during drought when atmospheric demand for water is increasing but upper soil water supply is declining? (2) As soils dry, does tree water extraction shift to deeper layers? (3) Can we predict *Q* based on tree size, stand basal area, or soil water content in the upper 1 m? Results will provide key insights into the regulation of ecosystem *T* by dominant trees as water availability changes and by concurrent dynamics of soil water extraction that can be used to inform models of tropical forest response to drought.

## Materials and Methods

### Study Area

The study was conducted at the ZF-2 Tropical Forestry Experimental Station (02°36′33″S; 60°12′33″W; ∼130 m elevation), within the Biological Reserve of Cuieiras in central Amazon. According to Köppen’s classification, the climate is type Af (wet tropical) with a short dry season that generally occurs between July and September (Alvares et al., 2014; Wu et al., 2016). The temperature ranges from 26.8 to 28.8°C but can reach up to 31°C. The predominant soil type in the plateau areas (terra-firme) is a dystrophic yellow latosol (Chauvel et al., 1992), a very clayey Oxisol. Clay contents increase from 59% at the surface to 73% at 1 m depth, with an average porosity of ∼0.58 m^3^ m^–3^. Wilting point to field capacity ranges from 0.27 to 0.41 m^3^ m^–3^ for the upper 1 m depth (Chauvel et al., 1992; Marques et al., 2004). This closed-canopy primary forest includes at least 245 species from 48 plant families with heterogeneous tree sizes, ages, and canopy heights (de Oliveira et al., 2008). Most of the crowns are globular in shape, with little direct radiation entering in lower strata. Although the height of individuals varies widely, more than 50% are between 14 and 25 m, with emergent trees reaching up to 50 m (de Oliveira et al., 2008). Estimates of tree age based on carbon isotopes for 20 species in the area range from 200 to 1,400 years (Chambers et al., 1998).

### Micrometeorological Variables

Micrometeorological variables were obtained from the 50 m K-34 tower managed by the Large-Scale Biosphere-Atmosphere Program (LBA) (Araújo et al., 2002). Precipitation, air temperature (T_air_), and relative humidity (R_H_) were measured by a rain gauge (EM ARG100, Environmental Measurements Limited, UK) and thermo-hygrometer (HMP45AC, Vaisala, Finland), respectively, in the open at 28 m height. Vapor pressure deficit (VPD) was derived from T_air_ and R_H_ using the Tetens formula (Tetens, 1930). All micrometeorological variables were collected at a 30-min time frequency.

### The Accumulated Daily Water Deficit

As in most humid tropical forests, precipitation events during the dry season are common. Thus, to define a dry period, we used the accumulated daily water deficit (ADWD; e.g., Santos et al., 2018) metric. Similar to the cumulative water deficit and maximum cumulative water deficit metrics (Malhi et al., 2009), the ADWD measures the duration and intensity of a drought using the cumulative precipitation from the previous 30 days (Santos et al., 2018). When precipitation is greater than monthly evaporative demand, the index returns zero. In situations where precipitation is less than monthly evaporative demand (in our case, ∼100 mm; Malhi et al., 2009; Santos et al., 2018), the index assumes negative values, starting with –1, and drought intensity is measured by the lowest value recorded during the analyzed period. To reinforce the delimitation of the dry period, we also compared the ADWD calculations to declines in soil water content and increases in VPD.

**FIGURE 1:**
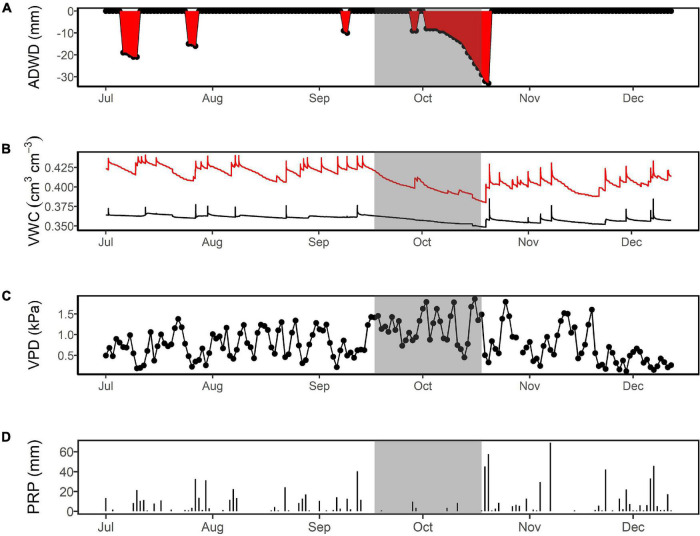
Characteristics of the 2018 dry season at ZF2 in the central Amazon (typically, July to December). The highlighted gray period is indicated by four metrics: **(A)** the accumulated daily water deficit (ADWD), which is used to describe the duration and intensity of the drought; **(B)** volumetric water content (VWC) at 15 cm (red line) and 75 cm (black line) depth, which indicates rates of water extraction; **(C)** vapor pressure deficit (VPD) as an explanatory variable of evaporative demand; **(D)** and precipitation, as a variable governing the volume of water entering the system.

The longest ADWD period in 2018 was identified from October 2 to 20, with a cumulative water deficit of 31 mm (Figure 1A). During this 2-week dry-down, the highest VPD values were observed along with the lowest soil water at 1 m, making ADWD a good metric to characterize drought intensity. We also included the previous 2 weeks (September 16–October 1) as they represented initial pre-drought conditions with relatively low rainfall and soil water and high VPD (Figures 1B–D). However, we discarded the last 4 days (October 17–20) because a series of significant rain events caused an increase in soil water and a drop in VPD (Figures 1B–D). Therefore, we defined the dry period as from September 16 to October 16, 2018 (shading in Figure 1).

### Species Selection

**FIGURE 2:**
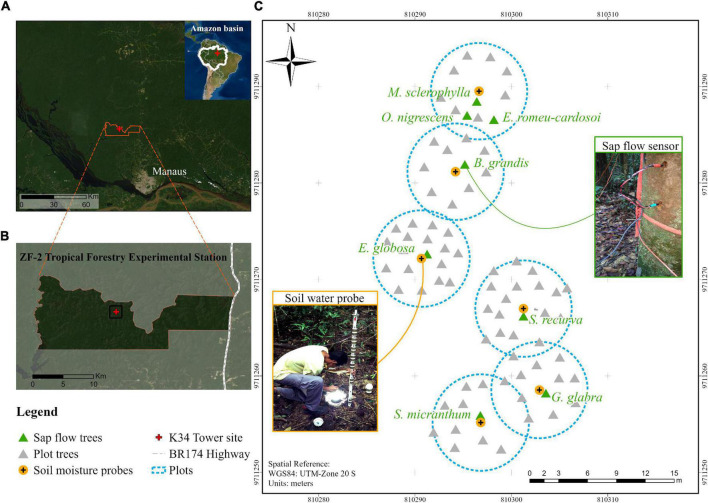
**(A)** Location of the study in the central Amazon at the **(B)** ZF2 forest research area north of Manaus, Brazil. **(C)** Spatial location of the sap flow and soil moisture instrumentation—dominant trees with installed sap flow sensors are labeled in green and associated soil water profile sensors are numbered in orange. The circular plot was delineated as a 5-m radius from the soil water sensor, and the diameter at breast height of all trees > 5 cm diameter within this plot were measured for scaling water use. The Large-Scale Biosphere-Atmosphere Experiment in Amazonia (LBA) K34 eddy covariance tower is labeled in red.

For this study, eight dominant canopy tree species were selected for sap flow monitoring, and six of these were additionally paired with soil water sensors to quantify water extraction (Figure 2 and Table 1). These species were selected because they are generally abundant in this forest stand (de Oliveira et al., 2008) and individuals were located close to one another and adjacent to the K-34 flux tower. We selected one individual tree per species. For each tree, we measured diameter at height 1.3 m from the ground (DBH) and height (HT) using a laser rangefinder (Trupulse, Laser Technology, Inc., United States), targeting the most emergent branch. We also estimated canopy area (CA) as the average of the canopy diameters measured from the forest floor in the north-south and east-west axes. To upscale *T*, we measured the DBH of all trees within six 10-m diameter plots centered on the six soil water profile sensors depicted in Figure 2. This included the adjacent sap flow tree and all other trees and lianas > 5 cm DBH within the plot. Species diversity was high with dozens of different tree species across the plots.

**TABLE 1:** Taxonomic and biometric information of sap flow sample trees indicating diameter at breast height (DBH), height (HT), crown area (CA), dye-based sapwood depth (SPD), and sapwood area (SA) and if trees were paired with fine root biomass collections or volumetric soil water probes.

Species	Family	DBH (cm)	HT (m)	CA (m^2^)	SPD (cm)	SA (cm^2^)	Fine roots	Soil water
*Buchenavia grandis* Ducke	Combreataceae	113.8	39.0	300	1.6	556	x	x
*Ocotea nigrescens* Vicent.	Lauraceae	71.0	28.0	257	9.3	1,721	-	-
*Scleronema micranthum* Ducke	Malvaceae	61.0	27.3	148	5.1	835	x	x
*Maquira sclerophylla* (Ducke) C.C. Berg	Moraceae	56.0	31.0	104	3.4	506	x	x
*Eriotheca globosa* Aubl.	Malvaceae	55.8	28.0	143	3.4	523	x	x
*Eschweilera romeu-cardosoi* S.A Mori	Lecythidaceae	36.0	22.0	93	3.5	333	-	-
*Goupia glabra* Aubl.	Goupiaceae	32.0	21.0	89	6.8	503	x	x
*Swartzia recurva* Poepp.	Fabaceae	29.8	20.5	37	2.5	202	x	x

### Sap Velocity, Sapwood Area, and Sap Flow

To calculate the water use or sap flow (*Q*) of each of the eight trees, we measured radial patterns of sap velocity (*v*_*s*_), sapwood depth (*SPD*), and sapwood area (*SA*) (Table 1). The *SPD* was measured in the field using a dye injection technique based on Goldstein et al. (1998). Briefly, a small ∼6.5 mm radius hole was drilled at a height of 1.3 m at a slight downward angle through the sapwood and into the heartwood and subsequently filled completely with dye (aqueous 0.5% acid fuchsin filtered to 0.2 μm) under slight pressure. The dye was retained in a 30 ml syringe reservoir held in place by its tip inserted through the outer bark and phloem tissue. Additional dye was added as needed. After 2–3 h, an increment core was collected ∼2–3 cm above each dye injection point and the conductive sapwood tissue depth was determined from the thickness of the xylem wood where dye was visible. From *SPD*, we calculated the total *SA* assuming that *SPD* was homogeneous across the trunk cross-section.

To measure *v*_*s*_, we used heat dissipation-type sensors with a 2-cm sensing tip (TDP-GS, PlantSensors, Australia; Granier, 1987). For each tree, the probes were installed at 1.3 m height at depths of 0–2, 2–4, 4–6, and 6–8 cm (depending on dye-estimated sapwood depth) within active sapwood to account for radial variation (Meinzer et al., 2001). Each sensor consists of a set of two temperature probes inserted into brass tubes which protect the probes and provide good conduction with the surrounding xylem. The two probes were separated by a vertical distance of 10–15 cm. The upper probe was continuously heated with 0.2 W, while the lower probe was used as a reference temperature. The difference in temperature (or voltage) between the two probes was used to calculate *v*_*s*_ (Oishi et al., 2016) as:


(1) $$v_{s}=a\,K^{b}$$

Where *v*_*s*_ is the sap velocity (g cm^–2^ s^–1^), a is a universal empirical calibration coefficient (0.0119), b is a constant (1.23), and *K* is the temperature difference between the two sensors. While studies have found that the calibration coefficient does not represent all species (e.g., Bush et al., 2010), it has proved appropriate in some tropical trees (e.g., McCulloh et al., 2007). In addition, for this study, we assumed the calibration coefficient was suitable for our trees. On the other hand, *K* is calculated by:


(2) $$K=\frac{△\,T_{m\,a\,x}}{△\,T}-1$$

where ΔT is the temperature difference between the heated and unheated probe, and Δ*T*_*max*_ is the temperature difference between the two probes at zero flow which is assumed to be reached on nights with low VPD and dew formation on foliage. Then, we upscaled *v*_*s*_ to whole tree-level sap flow (*Q*; g s^–1^) by summing *v*_*s*_ for each concentric ring of active sapwood depth (*i*):


(3) Q=∑i=1S⁢P⁢Dvsi×S⁢Ai

where *SA* is the sapwood area (cm^2^) at each sensor position into the sapwood (0–2, 2–4, 4–6, and 6–8 cm) until maximum depth (*SPD*) is reached (Table 1). Since some of the innermost sap flow sensors could partially extend into non-functional xylem, the integrated signal at that depth could be lower than what exists in the adjacent functional sapwood and may create some underestimation of sap flow at this depth, although error should be small since sap flow generally decreases with sap wood depth (Clearwater et al., 1999).

### Allometric Relationships and Upscaling Transpiration

To describe the response of *Q* (water use in L day^–1^) to the dry season, we focused our calculations on the dry period defined from ADWD, soil moisture, and evaporative demand. We used the beta coefficient from the linear regression of *Q* {[normalized by (x–min (x)]/[max (x)–min (x)]} as a function of the number of dry days (i.e., 1,2,3…n) to describe water use trends. Values > 0 demonstrate an increase in water use over the course of the drought and trends < 0 demonstrate a reduction in water consumption. We only used days with high evaporative demand, defined here as days with VPD > 1 kPa, excluding wet days or those with low evaporative demand and likely low water use.

Then, we upscaled *Q* to plot-level *T*. To do so, we first modeled tree-level *SA* as a function of DBH. We pooled data from our eight trees with additional allometry data collected by Aparecido et al. (2019) at the same site. In Aparecido et al. (2019), the relationship between *SA* and DBH was limited to trees with DBH < 40 cm. In our study, the DBH of individual trees within the plots ranged from 5 to 113.8 cm, with 5 trees having a DBH > 40 cm. To avoid excessively weighting outliers, we applied Cook’s method (Belsley et al., 1980; Cook and Weisberg, 1982) to detect potential outliers within the pooled data. We detected two outliers: *Buchenavia grandis*, which was the largest tree, and *Ocotea nigrescens*, which had the largest sapwood area (Table 1). Removing these two outliers resulted in the highest correlation coefficient of *SA* vs. DBH as compared to the total dataset or the dataset minus each outlier. The results were used to improve the Aparecido et al. (2019) allometric equation (Supplementary Figure 2). In this way, we had a method to estimate *SA* based on DBH. Second, we used our eight species with sap flow sensors to draw a relationship between *Q* and *SA* before calculating *Q* based on DBH. Then, we upscaled *Q* to plot-level *T* according to:


(4) $$T=\sum_{i=1}^{n}Q_{i}$$

Where *T* is the summed transpiration for all trees (n) within the 10 m diameter plot and *Q*_*i*_ represents the *Q* of each tree in the plot estimated based on the two allometric relationships: *Q* vs. *SA* and *SA* vs. DBH. Plot level *T* could then be directly related to plot level basal area. The six plots were centered on each of the soil moisture probes that were installed 1 m away from six of the sap flow trees (Figure 2). We compared this estimate of plot level *T* (Eq. 4) to measured sap flow of the dominant tree (Eq. 3) in each plot. Because *T* is at the plot level, we could then compare *T* to the water extraction measured by the soil water sensors.

### Volumetric Soil Water Content

For six of the eight sap flow trees, we measured volumetric soil water content (cm^3^ cm^–3^; Figure 2 and Table 1) using multi-frequency domain capacitance probes (FDC; EnviroSMART, Sentek Pty. Ltd., Stepney SA, Australia). The FDC probes were inserted into PVC access tubes located at 1 m distance from each tree. Initial installations reached 235 cm depth, however, poor upper soil contact with the access tubes due to compaction required careful reinstallations to 1 m depth. Soils from the initial installations were used for root assessment (described below). Each probe consisted of seven sensors centered at the vertical depths of 10, 20, 30, 40, 50, 70, and 100 cm. Results were recorded using a datalogger (model CR1000, Campbell Scientific Inc., Logan Utah United States) at 15-min intervals. Each sensor provides an integrated vertical measurement spanning ∼10 cm, e.g., the sensor at 10 cm represents the 5–15 cm layer. To improve accuracy of the soil moisture measurements, we performed a site-specific calibration (described in Supplementary Data).

### Fine Root Biomass

**FIGURE 3:**
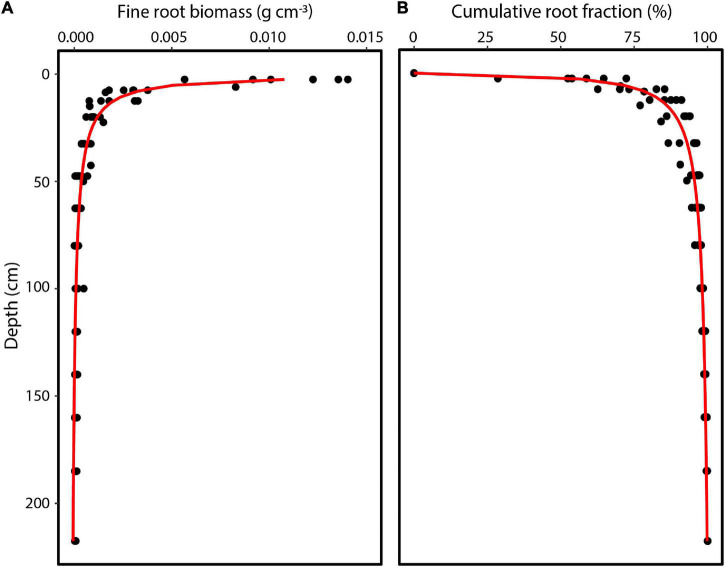
Vertical distribution of **(A)** fine root biomass and **(B)** cumulative root fraction in the 0–235 cm profile for an upland soil in a tropical monsoon forest in central Amazonia. Data represent 5–6 replicate locations.

In order to measure fine root biomass distribution, soil samples of a known volume were collected from the same locations as the FDC soil water sensors (Figure 2 and Table 1). Soil was excavated at progressively larger depth intervals, from an initial 5 cm intervals for the root-dense upper soil to a 35 cm interval at 200–235 cm. In the field, soils were stored in plastic bags to be transported to the Forest Management Laboratory at the National Institute of Amazonian Research. In the lab, roots were manually separated using steel mesh sieves of various diameters and tweezers. Only the roots with a diameter < 2 mm were retained, while black roots which had died and decayed were discarded (Vogt and Persson, 1991). The fine roots were placed in paper bags and dried at 65°C for more than 48 h until constant weight was obtained. Given the high diversity of species, we neither separate roots by species nor measure fresh root length. To describe fine root biomass distribution, we fitted a non-linear model to fine root biomass and cumulative fraction as a function of soil depth:


(5) $$Y=1-\beta^{d}$$

where Y is the cumulative root fraction at depth d and β is the root distribution coefficient (Gale and Grigal, 1987). Despite a good exponential curve fit of biomass to depth using the Gale and Grigal (1987) model (β = 0.8, *R*^2^ = 0.92), the cumulative root fraction between 20 and 50 cm was overestimated. Therefore, we tested a more complex Bleasdale yield-density model:


(6) $$Y=d\times {\left(a+b\times d^{c}\right)}^{-1/c}$$

where Y is the root fraction at depth d (Bleasdale and Nelder, 1960) and a, b, and c are fitted coefficients. This model provided a better fit to a depth of 235 cm (Figure 3: *R*^2^ = 0.90; *a* = 1.5024, *b* = 0.9859, *c* = 0.8520).

### Soil Water Extraction

Change in soil water content during the dry period was calculated as the difference in the initial and final volumetric soil water content during each 15-min interval for each of the six probes. Soil water extraction from the upper 1 m was then estimated as the daily sum of the change in water content for each layer. At depths without sensors (e.g., 60 cm), data were interpolated based on the adjacent sensors (at 50 and 70 cm). In this study, as in prior studies (Salati and Vose, 1984; Kunert et al., 2017), evaporation at the soil surface was assumed to be negligible since the dense forest canopy greatly limits solar radiation to the forest floor that would create the temperature gradient to drive evaporative water loss. In addition, the soil in the plateau is a well-structured and well-drained clay with fast infiltration, such that there is limited pooling of surface water. Thus, the change in soil water content over the defined dry period was primarily attributed to root water uptake. We also assume drainage losses are minimal given that high plant transpiration demand during the dry season would help offset downward hydraulic gradients. The minimal drainage assumption is also supported by Tomasella et al. (2008) who suggested negligible groundwater recharge flux from the K34 plateau during the dry season. Water extraction was not calculated following small precipitation events during the dry period due to precipitation moving into the upper soil layers.

To relate fine root biomass to soil water extraction up to 1 m depth, we used a non-linear Michaelis-Menten type regression:


(7) $$V=V_{m\,a\,x}\,\left(\frac{R_{d}}{K_{d}+R_{d}}\right)$$

where V is the extraction rate, R_d_ is the root density, and K_m_ is a rate constant. Since tree water extraction can shift to deeper depths during drying periods, we also fitted a logarithmic function of soil water extraction by soil depth to project potential water extraction to a depth of 2 or 3 m. The logarithmic fit allowed for an alternate estimate of plant water use for depths greater than 1 m. The logarithmic fit also provided an estimate of water extraction between a depth of 0 and 10 cm at the beginning of the dry period. At the end of the dry period, it is likely that uptake was limited at this layer. Thus, uptake was set to decline to zero at the surface.

### Data Analysis

Data were analyzed using R v. 3.0.2 (R Core Team, 2021). To compare total water extraction as the soil dried, we performed the Student’s *t*-test when the data were normal and the Wilcoxon test when data were not normal. *T*-tests were used to test differences between locations by depth. Normality was checked with the Shapiro-Wilk test. Lastly, we compared soil water extraction to plot-level *T* and upscaled using tree allometric relationships.

## Results

### Tree-Specific Water Use Trends During the Dry Period

**FIGURE 4:**
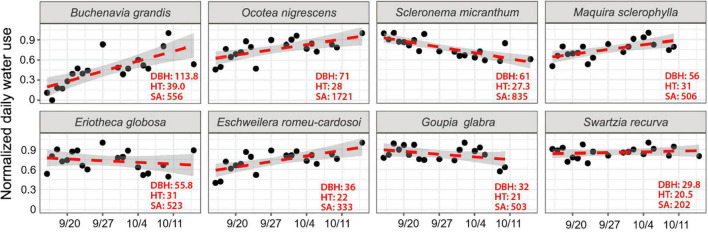
Normalized daily transpiration based on scaled radial patterns of sap flow from eight dominant canopy trees during the dry period from September 16 to October 16, 2018 for days with mean VPD above 1 kPa. Tree diameter (DBH; cm), height (HT; m) and sapwood area (SA; cm^2^) are indicated for each tree. The shaded area represents the 95% confidence interval.

For all individual trees, sap velocity was greatest in the outer 2 cm of sapwood, ranging from about 6 to 30 cm h^–1^ depending on species (Supplementary Figures 3A,B). For trees with deeper sapwood, sap velocity declined with depth, with minimum values by 6 cm depth and no sap flow at 8 cm depth. Sap velocity at different depths declined, remained flat, or increased over the course of the dry period depending on species. During the dry period, *Q* varied by species (Figure 4 and Table 2). Some trees increased water use in response to the drought, some remained unchanged, while others reduced water use during the drought. Trees that showed significant increases in daily water use as the dry period progressed (indicated by the slope of a linear relationship between time and normalized *Q*) were *Buchenavia grandis, Eschweilera romeu-cardosoi, Maquira sclerophylla*, and *Ocotea nigrescens.* Trees which showed a declining trend in daily water use during the same dry period included *Scleronema micranthum*, while *Eriotheca globosa, Goupia glabra*, and *Swartzia recurva* remained constant (Supplementary Tables 1A,B). There was a wide range in tree-specific water use, from ∼11 L day^–1^ for *S. recurva* to 190 L day^–1^ for *O. nigrescens* (Table 2). During this dry period, there was little effect of VPD on *Q* except for *S. recurva*, which increased water use on high VPD days (Supplementary Table 1 and Supplementary Figure 1). High VPD was also correlated to higher air temperatures (Supplementary Figure 1).

**TABLE 2:** Mean, maximum, and minimum (± *SD*) daily transpiration by species from September 16 to October 16, 2018 based on scaled radial patterns of sap flow.

Species	Mean (L day^–1^)	Max (L day^–1^)	Min (L day^–1^)
*Buchenavia grandis* Ducke	63.1 ± 8.3	85.1	45.4
*Ocotea nigrescens* Vicent.	190.4 ± 27.4	230.2	117.6
*Scleronema micranthum* Ducke	56.5 ± 9.5	69.4	36.8
*Maquira sclerophylla* (Ducke) C.C. Berg	30.9 ± 5.7	39.8	17.8
*Eriotheca globosa* Aubl.	26.7 ± 3.3	32.3	16.8
*Eschweilera romeu-cardosoi* S.A Mori	77.0 ± 10.7	93.8	50.3
*Goupia glabra* Aubl.	42.7 ± 6.3	49.9	29.0
*Swartzia recurva* Poepp.	10.9 ± 1.6	12.7	7.0

### Soil Water Extraction by Depth

**FIGURE 5:**
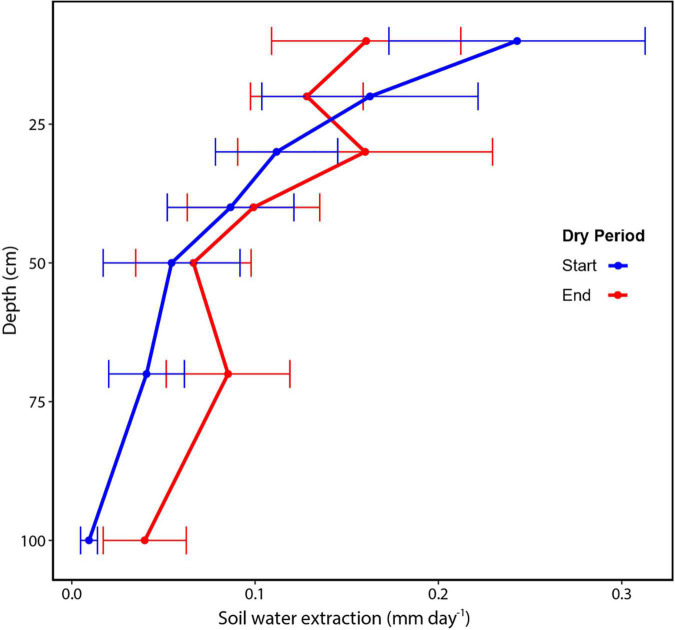
Soil water extraction by soil layer within the top 1 m soil for a tropical wet forest in central Amazonia. The start of the dry period (blue) was September 16 and the end of the dry period (red) was October 16, 2018. Values are averaged across 5–6 sensors at each depth, ± standard error.

**FIGURE 6:**
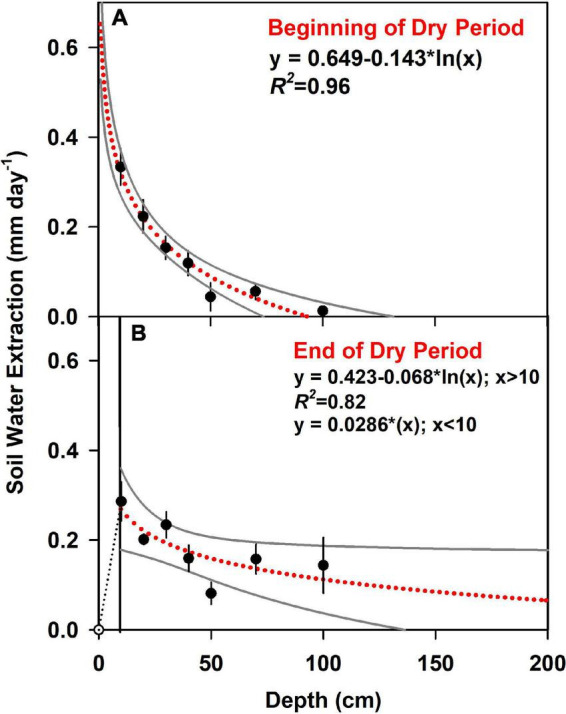
Soil water extraction is modeled as a logarithmic function of soil depth, with 95% confidence interval in gray. Points represent the average of actual measurements, with standard error bars. The beginning of the dry period **(A)** was September 16 and the end of the dry period **(B)** was October 16, 2018. As the soil surface was dry at the end of the drought, we assumed water extraction from 0 to 10 cm increased linearly with soil depth (reference Table 3 for integrated daily rates).

We determined soil water extraction based on changes in the volumetric soil water content over time at each depth. For the duration of the dry period, the average water extracted from the six plots from the upper 1 m was 0.86 ± 0.15 mm day^–1^, ranging from 0.67 to 1.01 mm day^–1^. We found temporal differences in the total water extracted from the top 1 m of soil (*t*-test; df = 5, *p* = 0.02), but shifts in extraction at individual depths were not statistically significant (*p* = 0.05), with *t*-tests for individual layers ranging from 0.065 to 0.34. At the beginning of the dry period, measured soil water extraction was 0.94 ± 0.09 mm day^–1^, but then decreased to 0.74 ± 0.07 mm day^–1^ by the end of the dry period (ref Figure 5). We also found differences in the vertical pattern of water extraction within the top 1 m of soil. Rates of water extraction declined with depth as both the root biomass declined. There was also a change in soil texture and extraction rates declined as sand content declined from ∼18% in the upper layer to 12% at 1 m depth (*R*^2^ = 0.93). At the start and end of the dry period, water extraction was consistently greatest in the shallow soil layers. However, the rate of extraction declined for the upper layers and increased for the deeper soil. Using the logarithmic function to extrapolate soil water extraction patterns beyond 1 m, we estimated that by the end of the dry period, ∼2.4 mm day^–1^ of soil water was extracted from the top 2 m (Figure 6 and Supplementary Table 2).

**TABLE 3:** Scaled transpiration for the dominant tree in each plot (Sap flow Tree *Q*; equation 3) and plot level transpiration for all trees within the 5 m radius plot (Plot *T*; equation 4), reference (Figure 2).

Plot	# Trees	Mean DBH ± *SD* (cm)	Total basal area (m^2^)	Sap flow tree *Q* (mm day^–1^)	Scaled plot *T* (mm day^–1^)
1	19	10.0 ± 7.0	0.22	0.29	1.9
2	10	15.8 ± 8.8	0.25	0.48	1.7
3	12	15.1 ± 16.3	0.44	0.38	2.2
4	18	15.9 ± 13.5	0.6	0.19	3.3
5	10	22.2 ± 33.5	1.18	0.21	3.2
6	9	16.0 ± 15.8	0.34	0.30	1.7

*Note large standard deviation (SD) for plot 5, which includes the largest tree in the research area.*

### Fine Root Biomass Distribution and Soil Water Extraction

**FIGURE 7:**
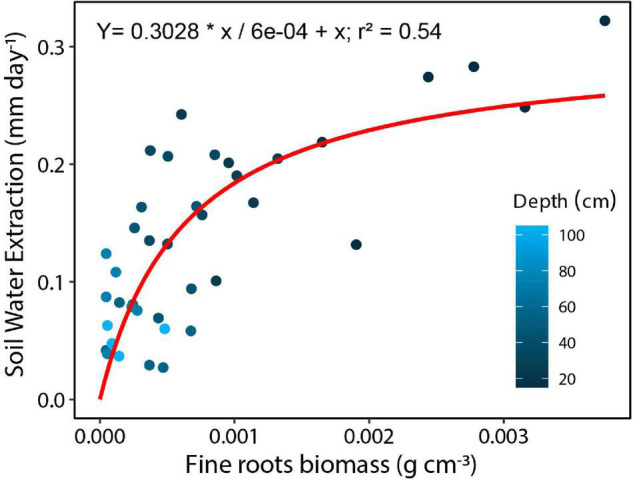
Soil water extraction by layer for the top 1 m of soil based on measured volumetric water contents (10, 20, 30, 40, 50, 70, 100 cm) as a function of fine root biomass within each layer for a tropical monsoon forest in central Amazonia. Darker circles are shallower depths.

Fine root biomass distribution measured for the top 235 cm of soil averaged to 1.19 kg m^–3^ and ranged from 0.97 to 1.37 kg m^–3^ by soil core location. Fine root biomass distribution exponentially declined with depth, with 45% of root biomass (range 29–60%) in the upper 5 cm. By integration, we found that 89% of the fine root biomass was distributed in the top 20 cm, 95% in the top 45 cm, and only ∼4% between 45 and 150 cm soil depth. Moreover, we found a significant relationship between fine root biomass and mean soil water extraction during the dry period, with fine root biomass explaining 59% of the variation of vertical soil water extraction (Figure 7; Equation 6: V_max_ = 0.25, *K*_m_ = 0.00042, *R*^2^ = 0.59). There was no relationship between root biomass and the adjacent sap flow tree diameter (*R*^2^ = 0.22) or root biomass and plot level basal area (*R*^2^ = 0.09).

### Plot-Level Transpiration and Soil Water Extraction

**FIGURE 8:**
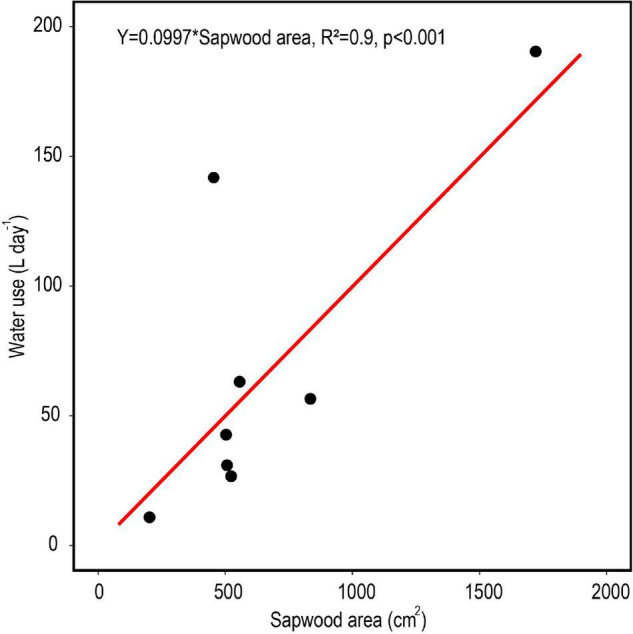
Average daily tree water use based on radial patterns of sap flow in relation to total sapwood area for eight trees in central Amazonia during a month-long dry period. The red line represents a linear model with the intercept set to zero.

**FIGURE 9:**
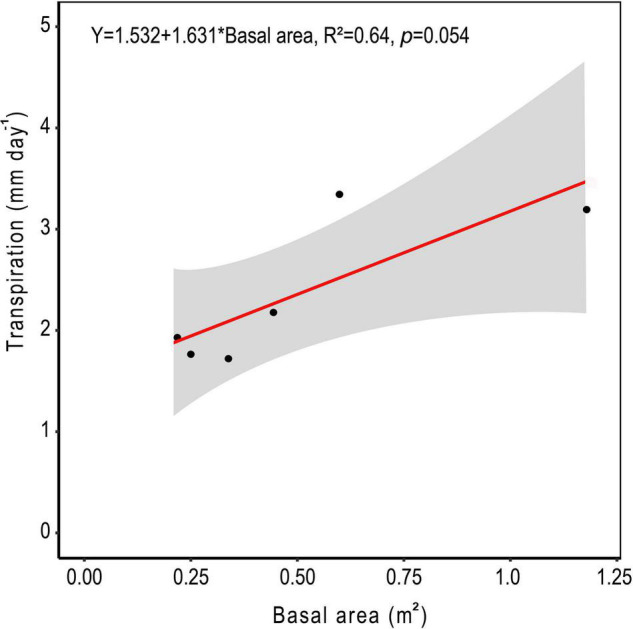
Average daily water use for all trees within each 10 m diameter sample plot based on the allometric scaling of tree basal area in central Amazonia. The shaded area represents the 95% confidence interval.

**FIGURE 10:**
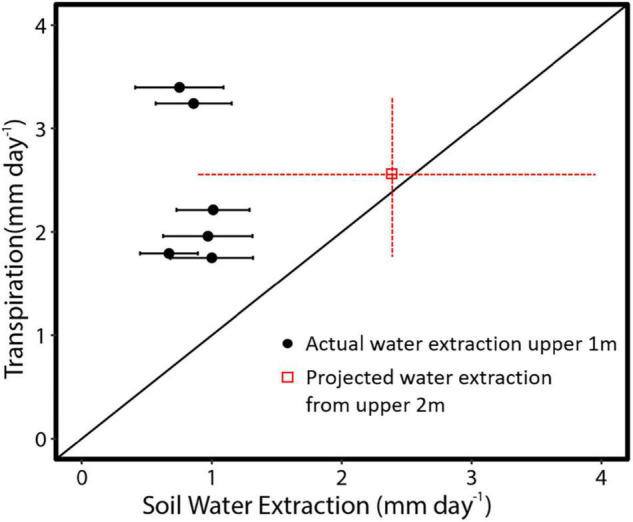
Average daily dry season transpiration for six adjacent plots in the central Amazon in comparison to mean measured or projected (Table 3) soil water extraction rates. Projected water extraction from the upper 2 m is based on a logarithmic fit (Figure 6). Black line is the 1:1 line. Red error bars show the range of transpiration and confidence intervals of projected water extraction from the upper 2 m of the soil profile.

The relationship between measured *SA* and DBH in this study was used to improve the allometric equation of Aparecido et al. (2019) (Supplementary Figure 2). Moreover, *SA* had a strong positive linear relationship with tree *Q* rate during the dry period (Figure 8; *R*^2^ = 0.91). By application of these relationships across trees within a plot, plot level basal area could be used to derive rates of plot level *T* (Figure 9), where increasing basal area (0.22–1.2 m^2^) explained 64% of the increase in plot *T*. Water use of the single sap flow tree in each plot (*Q*) scaled to projected canopy ground area (Table 1) indicated that single tree represented 6–28% of plot level water use (Table 3). Greater daily water use per unit ground area of the sap flow trees resulted in a reduction in whole plot level *T* (*R*^2^ = 0.59; Supplementary Figure 4). Plot level *T* was significantly greater than measured water extraction from the upper 1 m of the soil profile. However, projected water extraction from the upper 2 m profile was in the same range as plot level *T* (Figure 10).

## Discussion

In this study, we linked tree water use to soil water availability and extraction patterns, root biomass distribution, and tree allometry to better understand drought-driven shifts in depth of water uptake. Even so, the hyperdiversity within tropical forests can confound simple size-based allometric scaling of transpiration due to species-specific variation in hydraulic strategies and water use. Adequate consideration of the range of spatiotemporal responses of stand level transpiration to drought can provide a framework for projecting water use based on measured ecosystem traits, such as soil properties, tree demographics, tree size, and dynamics of tree hydraulic sensitivity and resistance, including deeper water extraction, phenology, capacitance, or hydraulic redistribution. Even with such complexity, our results provide key information on the regulation of transpiration by dominant trees as water availability changes, along with the simultaneous dynamics of soil water extraction that can be used to inform mechanistic models that project tropical forest responses to drought.

### Sensitivity of Sap Flow to Drying

Our first objective was to assess water use for different co-occurring canopy trees as atmospheric demand increases and upper soil water declines. We found that some individuals increased transpiration rates during the month-long drought, while others decreased transpiration rates. There were also differential and dynamic radial patterns of sap flow during the dry period (Supplementary Figure 3), likely due to differential depth of root water extraction, timing and magnitude of transpiration, and stem water storage, use, and refilling—topics that are currently under active investigation. Results highlight divergent hydraulic strategies. Specifically, increases in transpiration indicate that these individuals have sufficient water supply and hydraulic conductivity to meet increased atmospheric demand. An important consideration to temporal water demand and supply dynamics is tree size. Generally, we expect that taller trees that rise above the canopy experience greater water use due to greater exposure to solar radiation (Kunert et al., 2017), and that larger diameter trees can have greater internal water storage capacity to help fulfill that demand (Scholz et al., 2011). Indeed, we found that the emergent *B. grandis*, which had the greatest height and diameter, also had the steepest rate of increase in daily whole tree sap flow (*Q*) during drought.

We found a positive relationship between tree size and water use across the entire range of sampled sap flow trees, similar to that reported in other tropical forest studies [e.g., Meinzer et al., 2001 (Panama), Kunert et al., 2017 (Central Amazon)]. However, at narrower diameter ranges, this relationship can break down due to differences in sapwood depth and hydraulic strategy. For example, trees larger than 30 cm diameter had a wide range of sapwood area which was not well linked to diameter (Supplementary Figure 2) or tree water use, i.e., despite have a 60% greater diameter, the total daily *Q* for *B. grandis* was just one third that of the smaller *O. nigrescens—*a result of the much lower *SA* of *B. grandis*. Our field observations indicate that large *B grandis* are often decayed and hollow inside. Similarly, in another nearby study at ZF2, there was no relationship between diameter and water use for trees > 40 cm (Kunert et al., 2017). In contrast, for canopy tree species in Panama, there was a tighter relationship between *SA* and diameter, and deep sapwood was more prevalent for the larger trees (Meinzer et al., 2001). While broad scaling patterns are evident, the higher resolution differences in relationships between DBH, *SA*, and *Q* suggest that other phenological, structural, and physiological characteristics such as wood density, xylem vessel size, stem hydraulic conductivity, and capacitance are also important for determining plant water use (e.g., Bucci et al., 2008; Santiago et al., 2018). Across 27 co-occurring canopy trees in Panama, sap flux density dramatically declined with tree size, although larger trees often have greater *SA*, which could offset the reduced rates and maintain high total tree water use (Meinzer et al., 2001).

Tree water use and xylem transport rates depend on sapwood water storage or capacitance, which increases with *SA* and can vary with wood physical properties, e.g., declining with wood density (Meinzer et al., 2003). Leveraging capacitance during dry periods requires continued access to soil water. Thus, deeply rooted trees may maintain high transpiration rates during drought (Lee et al., 2005; Baker et al., 2008) when buffered by diurnal use and refilling of stored water in stem or in the upper roots and soils via hydraulic redistribution. Indeed, the importance of stem water storage (e.g., Goldstein et al., 1998; Scholz et al., 2008; Yan et al., 2020) and hydraulic redistribution has been shown to be a significant component of diurnal and seasonal water use during dry periods (e.g., Lee et al., 2005; Neumann and Cardon, 2012). Because both processes would provide a buffer for net daily decline in upper soil water availability, apparent upper soil water stress can be delayed.

Alternately, decreases in *Q* indicate that these individuals are sensitive to the drying of near surface soils. After B. *grandis*, the next four largest trees were co-dominant with similar heights. Still, drought-responses differed. There was an increase in *Q* for two trees, but a decline for the other two. Moreover, two of the three smallest trees had a flat or declining rate of *Q*. The differential sensitivity to drought is likely related to their hydraulic strategy, including stomatal sensitivity to VPD, phenology or leaf loss, capacitance or rooting depth, and ability to extract soil water held at high tensions—traits which may be species-specific. Because we found that even a week without precipitation was sufficient to reduce much of the plant available water near the soil surface, 0–40 cm deep, we might expect that some shallowly rooted species tolerate the low leaf water potentials necessary to continue to extract water and maintain or increase *Q* during short dry periods. Meanwhile, others that cannot tolerate low water potentials would decrease *Q*. One of the smallest trees, *Eschweilera romeu-cardosoi*, displayed large increases in *Q* during the dry period. This species could meet increased water demands if it either tolerates low leaf water potentials, is deeply rooted, or, alternately, has more extensive lateral rooting that may be able to access pockets of higher water content spatially across the landscape. Linking water use to root biomass by species was not possible since we only assessed sap flow in one individual per species and the adjacent root biomass distribution consisted of many species. Root distributions are remarkably diverse and, in some cases, can extend long distances across the landscape to access resources where available, e.g., a neotropical fig (*Ficus schultesii*) in a Peruvian floodplain was found to have a 102 m long lateral surface root (Silman and Krisel, 2006).

### The Forest Shifts to Deeper Soil Water Extraction as Soil Dries

Our second objective was to track shifts in the depth of water extraction as soils dry. Yet, the ability to extract soil water at deeper depths is constrained by rooting depth. We found that fine root biomass was greatest near the surface. Our field observations indicated a root mat at the surface (<2 cm depth) intermixed with a minimal leaf litter layer. After that, root density declined rapidly with depth. These results are not surprising and agree with most studies in the Central Amazon. For example, Chauvel et al. (1992) showed that root length was concentrated in the superficial soil horizons, but then decreased rapidly with depth, i.e., 70 km m^–3^ at 0–30 cm depth while deeper layers (1–6 m) were just 3.5–6.5 km m^–3^. Ferreira et al. (2002) showed that 74–93% of fine root biomass was found in the top 20–40 cm, respectively, and Noguchi et al. (2014) showed that 89% of the fine root biomass in the plateau was concentrated in the first 20 cm, in agreement with our results. The higher concentration of biomass of fine roots near the surface is likely due to the higher levels of organic matter, litter decomposition, and nutrient cycling in addition to the physical structure of the soil (lower density, higher porosity, and higher concentration of sand in the upper soil). Observations at a nearby site with minirhizotrons reported that root length production and mortality were also much greater in upper soils and more sensitive to precipitation events (Cordeiro et al., 2020). Since these soils are generally nutrient poor, primarily low in phosphorus (Lugli et al., 2020; Newman, unpublished data), root distribution at the surface can convey competitive advantage for interception of nutrients released from surface litter decomposition.

Furthermore, high root density at the surface would allow for rapid uptake of precipitation, which could be important to alleviating drought stress during periods when the frequency or magnitude of precipitation is limited. During wet periods, upper roots would also provide benefit for nutrient uptake prior to the rapid infiltration and movement of water through the well-structured soil. While we did not assess surface root water uptake, we projected an exponential increase in uptake toward the surface. The importance of this finding is that despite the high concentration of fine roots near the surface, it is the deeper roots which will become critical for sustaining plant function during extended dry periods. The trees could be using shallow roots for growth and deep roots for maintenance/survival, as modeled for arid and semiarid biomes (Ryel et al., 2008).

We found that at the stand-level, the forest generally shifted to deeper soil water sources. In another study at K34, Broedel et al. (2017) reported a significant increase in the depth of water extraction during the evolution of a drought. This pattern has also been found in other forest types with dry seasons (Nepstad et al., 1994, 2007; Hodnett et al., 1995; da Rocha et al., 2004; Warren et al., 2005; Bruno et al., 2006; Tomasella et al., 2008), including the southern Amazon (Negrón-Juárez et al., 2007). Hodnett et al. (1995) showed that only 80 mm of water between 2 and 3.6 m depths were available in the soil to plants. Even so, these deep layers provide an additional volume of water to the forest during dry periods as a way to compensate for the higher evaporative demand, reflections of low levels of precipitation, high air temperatures and VPD, and low air and soil humidity. Our data demonstrate that at the plot-level, there was an increase in total water extraction and water extraction with soil depth (Supplementary Table 2). Since soil nutrients are concentrated toward the surface, this pattern of water extraction has direct implications for seasonal patterns of nutrient uptake for this site and other forests with similar seasonal shifts in water uptake to deeper depths.

Soil water availability has been shown to be limited for the Oxisols of the central Amazon, with only ∼18% availability of total water between field capacity and the permanent wilting point in the upper 1 m (Ferreira et al., 2002). Given the high clay content present at our site (Corrêa, 1984; Chauvel et al., 1992; Marques et al., 2004), we would expect that in an extended dry period, root extraction exhausts the soil water pool of the top 1 m and that deeper soil water reserves must be tapped to sustain transpiration. If deeper soil water is not available because the soil is dry or there is a lack of roots, transpiration must decline. In the more seasonal eastern Amazon, tree water use was reduced after a 21-day dry period characterized by low soil moisture and high VPD, indicating a lack of water extraction from deeper depths (Brum et al., 2018). At our site, upper soil water content substantially declined during the month-long dry period, likely reaching the point where water was no longer available for plant uptake. For example, at 15 cm depth (Figure 1B), water content declined from ∼43 to 38% by the end of the drought. Earlier work in a nearby plateau that modeled van Genuchten soil water release parameters indicated that water content at the wilting point (1,500 kPa) was ∼37% for the 10–20 cm layer (Ferreira et al., 2002), suggesting a substantial loss of water available for extraction. Water balance data from a deep pit at the site also indicated a lack of water availability in the upper 1 m by the end of the dry period (de Oliveira, 2020). Since dry season transpiration can be maintained at ∼3 mm day^–1^ at the site (Negrón-Juárez et al., 2007), the lack of water availability in the upper layers toward the end of the dry period illustrates the necessity of progressively deeper water extraction to fulfill the transpiration demand of the trees. Since we observed divergent trends in *Q* rates during dry conditions, we expect that species which were heavy water users (having an increased rate and daily peak of *Q*) were important in driving shifts to greater plant-water-uptake depth. The spatial patterns of soil water extraction across the landscape should thus be reflected by the species trait distributions and their underlying effective rooting depths (e.g., Chitra-Tarak et al., 2021).

### Discrepancy Between Stand Level T and Soil Water Extraction

Our last objective was to predict *T* based on tree sizes, stand basal area, and soil water extraction in the top 1 m. Specifically, we used allometric equations to scale tree size into stand-scale *T* and then compared to total soil water extraction. There are several reasons that could lead to a discrepancy between these methods. One reason is related to the assumptions we make to estimate stand-level *T*. Essentially, we are asking, can we use diameters of trees in the stand to estimate *T*? This question has previously been posed by others. For example, Meinzer et al. (2001) found that diameter alone accounted for more than 90% of the variation in both maximum and total daily sap flux density in the outer 2 cm and that sapwood area scaled strongly with diameter. In contrast, we found large variation in sapwood depth for the larger individuals in our study such that tree size accounted for just 60% of variation in sapwood area. In addition, we found little relationship between maximum sap flux density in the outer sapwood and tree size, but were still able to leverage the radial patterns of sap flow in order to estimate *Q* in our study that could then be applied to estimate plot *T*. Other research has shown that stand-level, time-averaged scaling overlooks species-level differences and fluctuation in environmental conditions (Anderson-Teixeira et al., 2015). This is supported by species-specific differences in *Q*, and hence, the need for outlier detection when performing allometric relationships between DBH, SAP, and T. That is, if we were to integrate a longer timeframe or contrasting environmental conditions over different species, we would expect our allometric relationships to change. This variability is highlighted in Meinzer et al. (2005) who illustrated that different, non-linear (2–4 parameter) scaling relationships are required both within and between angiosperm and coniferous tree groups.

Another reason for the discrepancy between scaling methods is that there are inherent differences between *Q* estimated via sap flux sensors and *T* (as scaled *Q*) estimated via water extraction patterns in the top 1 m of soil. Practically speaking, the point at which these water fluxes are measured and how measurements are scaled are different. Scaling thermal dissipation probe voltage output to sap flux density relies on accurate species-specific calibration equations which are not easily or often measured, thus leading to potential bias in scaling using the standard equation (e.g., Bush et al., 2010). Moreover, sap flux-based estimates at the tree-level integrate across the soil depths from which water is accessed, which may be greater than 2 m depth for some trees. Measurements of soil water extraction, on the other hand, are limited to the depths at which soil water is measured. Although we aimed to overcome some depth limitations by extrapolating soil water extraction patterns to 2+ m depth, tree water access at even greater depths is likely given the presences of some roots (e.g., Negrón-Juárez et al., 2020), albeit at very low densities, and the necessity for access to plant-available water. Since stand-level *T* estimates were consistently greater than soil water extraction estimates and since we have seen a general shift toward deeper soil water during the dry period, it is likely that soil water extraction extended beyond the 2 m depth during the study period and would necessarily be deeper than 2 m under a more extended drought. Indeed, a concurrent study at the site using data from a deep soil pit indicated a decline in soil water content at 2.4 m and below, which may be indicative of deeper root water uptake (de Oliveira, 2020).

## Conclusion

Our results provide field-based empirical evidence for a dependence on deep soil water sourcing due to drought-driven shifts in tree water use in the Central Amazon. Such findings are imperative given the sensitivity of tropical forests to changes in climatic conditions and considering that in the past, the humid tropics have only experienced extreme water stress periodically (often compounded during El Niño events), a pattern that is likely to change in the future. Here, we show that even a month of drought is sufficient to warrant a transpiration response. We found that differential transpiration patterns for co-occurring canopy species, the major water users contributing to stand-level signals, reflects divergent hydraulic strategies. Even though most of the tree root biomass is found near the soil surface, it is an increased dependence on deep soil beyond the top 1 m or more that sustained transpiration for some species and, more generally, at the forest stand-level. Moreover, discrepancies between sap-flux-based allometric estimates of *T* and total soil water extraction highlights challenges when scaling tree-based water use to stand-level estimates of ecosystem water use at higher spatial scales, and thus provides impetus for a holistic assessment of contributing soil, tree, and environmental components in a modeling framework. Understanding how different species respond to and cope with drought and quantifying this difference in terms of transpiration fluxes is critical as a first step to upscaling forest dynamics and understanding validity of model representation and community-scale generalizations. We expect that these findings will inform and help better constrain mechanistic stand, watershed and Earth System models that are used to project functional responses of tropical forests to drought.

## Data Availability Statement

The raw data supporting the conclusions of this article will be made available by the authors, without undue reservation. Sapflow and soil water datasets will be archived and available at: https://ngt-data.lbl.gov/.

## Author Contributions

JW planned and designed the experiments. GS, BG, VM, and JW performed the experiments. GS, CW, BG, VM, and JW analyzed the data. GS, CW, BG, and JW wrote the manuscript. All authors contributed to logistics, fieldwork, analysis, funding, or improved the manuscript.

## Conflict of Interest

The authors declare that the research was conducted in the absence of any commercial or financial relationships that could be construed as a potential conflict of interest.

## Publisher’s Note

All claims expressed in this article are solely those of the authors and do not necessarily represent those of their affiliated organizations, or those of the publisher, the editors and the reviewers. Any product that may be evaluated in this article, or claim that may be made by its manufacturer, is not guaranteed or endorsed by the publisher.
