# Supplementary material for: Dry Season Transpiration and Soil Water Dynamics in the Central Amazon
Source: Front Plant Sci. 2022 Mar 24;13:825097. doi: 10.3389/fpls.2022.825097 (PMC8987125; doi:10.3389/fpls.2022.825097)
Supplement: Supplementary file 1 [file Data_Sheet_1.docx]

**Supplementary Data**

**Dry season transpiration and soil water dynamics in the Central Amazon**

Spanner, G.C. et al., 2022, *Frontiers in Plant Science*

**Methods**

*Site specific calibration of frequency domain capacitance (FDC) soil moisture sensors*

The output of the FDC sensors is a dimensionless frequency that is converted using a normalization equation and then applied to a soil type-specific calibration equation by

| $\theta=(\frac{f-C}{A})^{\frac{1}{B}}$ | (5) |
| --- | --- |

Where f is the scaled frequency sensor output; θ is the volumetric water content (m^3^ m^-3^); A, B and C are the empirical coefficients of the equation. We initially used a calibration equation for a heavy cracking clay soil in Australia (‘Warren’ clay type, Sentek Calibration Manual, Sentek Pty Ltd), with calibration coefficients: A = 0.0254, B = 1, C = -0.150. This calibration resulted in reasonably, but slightly less absolute θ than other estimates at the site, thus we performed a site-specific calibration. Negrón-Juárez et al. (2020) also found that without a site-specific calibration TDR based moisture measurements at the site were substantially in error.

Since the primary focus of this study was to examine relative changes in soil water content, errors in the absolute calibration of the FDC sensors had minor impact on estimates of daily extraction rates. Even so, we adjusted the default factory calibration based on soil texture and using a site-specific calibration. For calibration, two access tubes were installed and allowed to equilibrate for four months, then a vertical FDC sensor was inserted into the tube and the volumetric water content using the ‘Warren’ type heavy, cracking clay (Sentek Calibration Manual; Sentek Pty Ltd 2001) was recorded. Immediately after inserting the FDC sensor, 5 cm tall metal Kopecky rings were carefully pushed vertically into the soil adjacent to the access tube. Each ring was carefully excavated, excess soil was cleaned off using a knife, then they were immediately wrapped in plastic for subsequent wet and dry gravimetric measurements. Based on these weights, volumetric water content was calculated as the mass of water divided by ring volume. Two replicate samples with seven depths were collected along either side of the tube down to ~60 cm. Some animal burrows and soil compaction affected several of the measurements, and two samples were identified as outliers and removed based on their interquartile range. The calibration regression between actual and FDC θ (R^2^ = 0.71) was then used to calculate *f* of the capacitance sensors from equation 5. Data were then used to back calculate a new calibration equation with coefficients A = 0.03154, B = 1, C = -0.6349, which can be used to convert *f* to VWC. There was one layer, 45-55 cm, that showed distinct physical differences from other layers above and below. This included lower bulk density (1.01 +/- 0.02 g cm^-1^ as compared with 1.18 +/- 0.04 g cm^-1^ for other layers in the upper 1 m) and a siltier texture (22% silt as compared with 4 - 6% for other layers in the upper 1 m) and a lower clay content (64% as compared with 80% for adjacent layers). Texture was quantified by the pipette method at the Soil and Plant Analysis Laboratory (LASP) of Embrapa Amazônia Ocidental, Manaus, Brazil. The differences in soil properties in the 45-55 cm layer result in greater macroporosity and water availability, which has been previously identified by other studies in the area (e.g., Tomasella and Hodnett, 1996; Broedel et al., 2017). For this layer, the heavy clay calibration resulted in low estimates of water content. As such, for the 45-55 cm layer we used the manufacturer’s default calibration (A = 0.1957; B = 0.404; C = 0.02852).

**References**

Broedel, E., Tomasella, J., Cândido, L. A., & von Randow, C. (2017). Deep soil water dynamics in an undisturbed primary forest in central Amazonia: Differences between normal years and the 2005 drought. *Hydrol. Proc.* 31(9), 1749–1759. <https://doi.org/10.1002/hyp.11143>

Tomasella, J., Hodnett, M.G. (1996). Soil hydraulic properties and van Genuchten parameters for a oxisol under pasture in central Amazonia. *In:* J.H.C. Gash et al. (Editors), *Amazonian Deforestation and Climate*. J. Wiley and Sons, New York, pp. 101–124.

**Results**

**Table S1a, b**. Linear regressions between normalized whole-tree sap flow (*Q)* and time during the seasonal dry period for each sap flow tree that highlight differential tree response to drying conditions (reference Figure 3); and linear regressions between daily *Q* and VPD during the dry period showing minimal relationship - together these data suggest soil moisture (or lack of precipitation) may provide more control on *Q* than VPD.

| Water use ~ Days of drought |  |  |  |
| --- | --- | --- | --- |
| **Species** | **Equation (normalized data)** | **R²** | **p-value** |
| *Swartzia recurva* Poepp. | 0.828 + 0.002x | -0.03 | 0.518 |
| *Goupia glabra* Aubl. | 0.909 - 0.008x | 0.77 | 0.138 |
| *Scleronema micranthum* Ducke | 0.971 - 0.019x | 0.58 | < 0.001 |
| *Eriotheca globosa* Aubl. | 0.793 - 0.006x | >0.01 | 0.32 |
| *Buchenavia grandis* Ducke | 0.099 + 0.035x | 0.6 | < 0.001 |
| *Maquira sclerophylla* (Ducke) C.C. Berg | 0.608 + 0.014x | 0.33 | 0.006 |
| *Ocotea nigrescens* Vicent. | 0.585 + 0.017x | 0.36 | 0.003 |
| *Eschweilera romeu-cardosoi* S.A Mori | 0.542 + 0.018 | 0.41 | 0.001 |
| Water use ~ VPD |  |  |  |
| **Species** | **Equation (L day^-1^)** | **R²** | **p-value** |
| *Swartzia recurva* Poepp. | 10.039 + 1.332x | 0.38 | 0.003 |
| *Goupia glabra* Aubl. | 45.126 + 0.912x | -0.05 | 0.74 |
| *Scleronema micranthum* Ducke | 66.576 - 3.137x | -0.03 | 0.51 |
| *Eriotheca globosa* Aubl. | 30.522 - 1.742x | -0.02 | 0.48 |
| *Buchenavia grandis* Ducke | 50.219 + 9.345 | -0.009 | 0.37 |
| *Maquira sclerophylla* (Ducke) C.C. Berg | 26.986 + 5.307x | 0.12 | 0.08 |
| *Ocotea nigrescens* Vicent. | 166.04 + 26.73x | 0.06 | 0.14 |
| *Eschweilera romeu-cardosoi* S.A Mori | 70.572 + 8.220x | 0.02 | 0.25 |

**Table S2**. Daily soil water extraction predicted by logarithmic functions fit to the upper 1 m soil water extraction patterns for the beginning and end of the dry period, September 16 and October 16 respectively (reference Figure 5).

|  | **Beginning of Dry Period** | | | **End of Dry Period** | |  |
| --- | --- | --- | --- | --- | --- | --- |
| Soil Depth | Soil water extraction | 95% CI Range | | Soil water extraction | 95% CI Range | |
| (cm) | (mm day^-1^) | (mm day^-1^) | | (mm day^-1^) | (mm day^-1^) | |
|  |  |  |  |  |  |  |
| 0 – 10 | 0.46 | 0.43 | 0.49 | 0.14 | --- | --- |
| 0 – 50 | 1.16 | 0.96 | 1.36 | 0.93 | 0.75 | 1.12 |
| 0 – 100 | 1.34 | 0.99 | 1.77 | 1.58 | 1.03 | 2.17 |
| 0 – 200 | --- | --- | --- | 2.42 | 1.07 | 3.99 |
| 0 – 300 | --- | --- | --- | 2.9 | 1.07 | 5.61 |

**
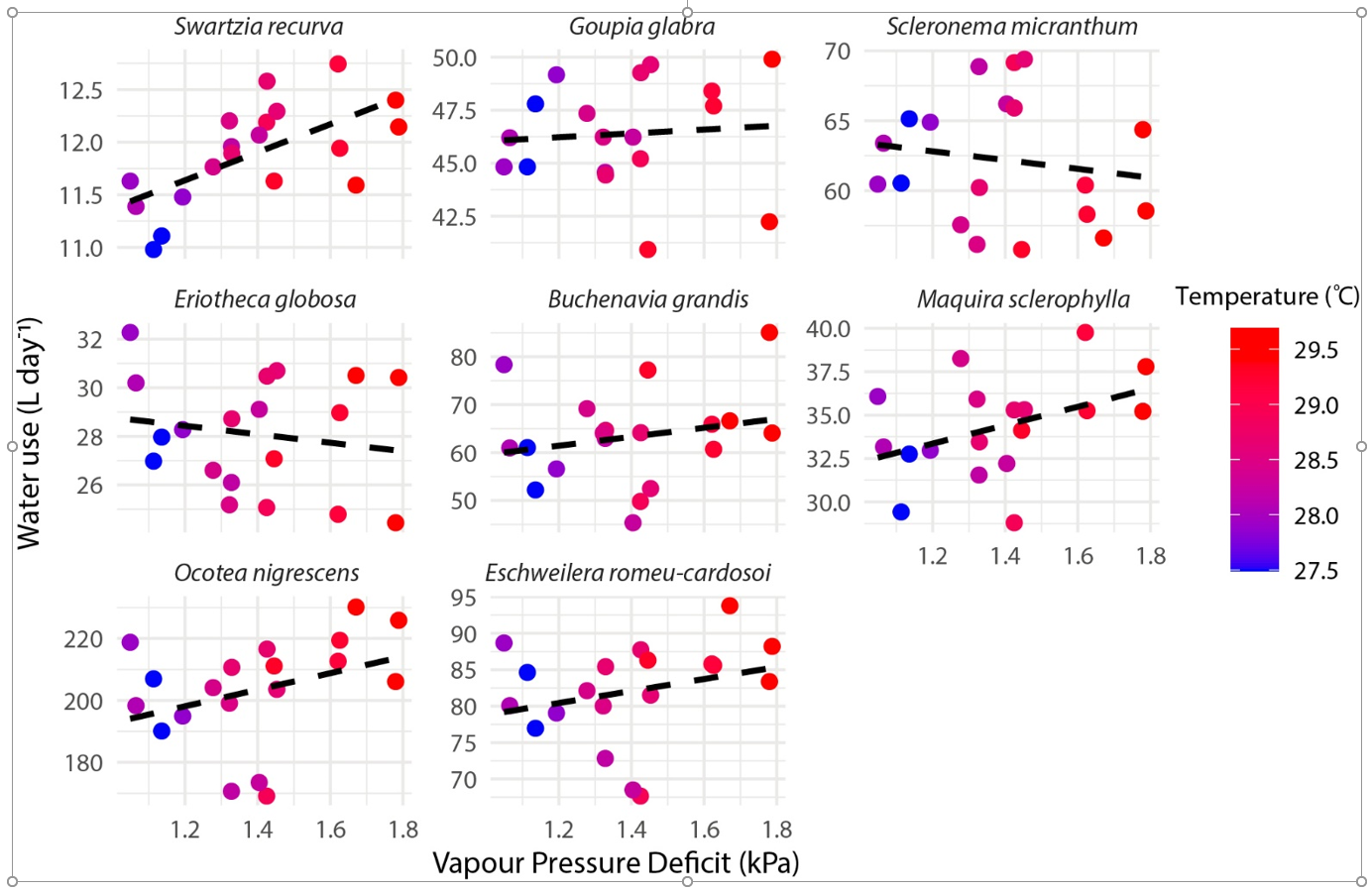
**

**Figure S1.** Linear regressions between whole-tree sap flow (*Q*) and VPD during the dry period for each sap flow tree. Color intensity indicates air daytime air temperature. Regression parameters shown in Table S1.


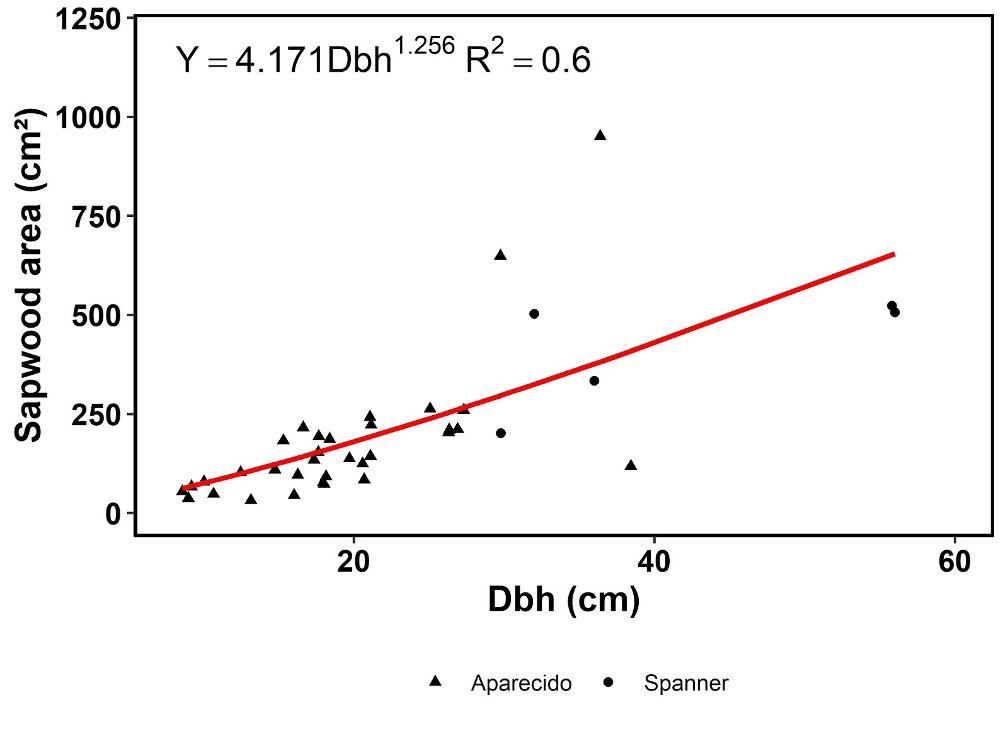


**Figure S2.** Scaling relationship for sapwood area based on diameter at breast height for trees in central Amazon. Data points from Aparecido et al. (2019) (triangles) and this study (circles). Two trees were excluded as outliers, with DBH/SA: 114/556 and 71/1721, putting them well off this graph, and which, if they were included in the regression significantly reduced the R^2^ (see main text). The wide range in their values indicates a research need for additional data beyond the scale of this figure.


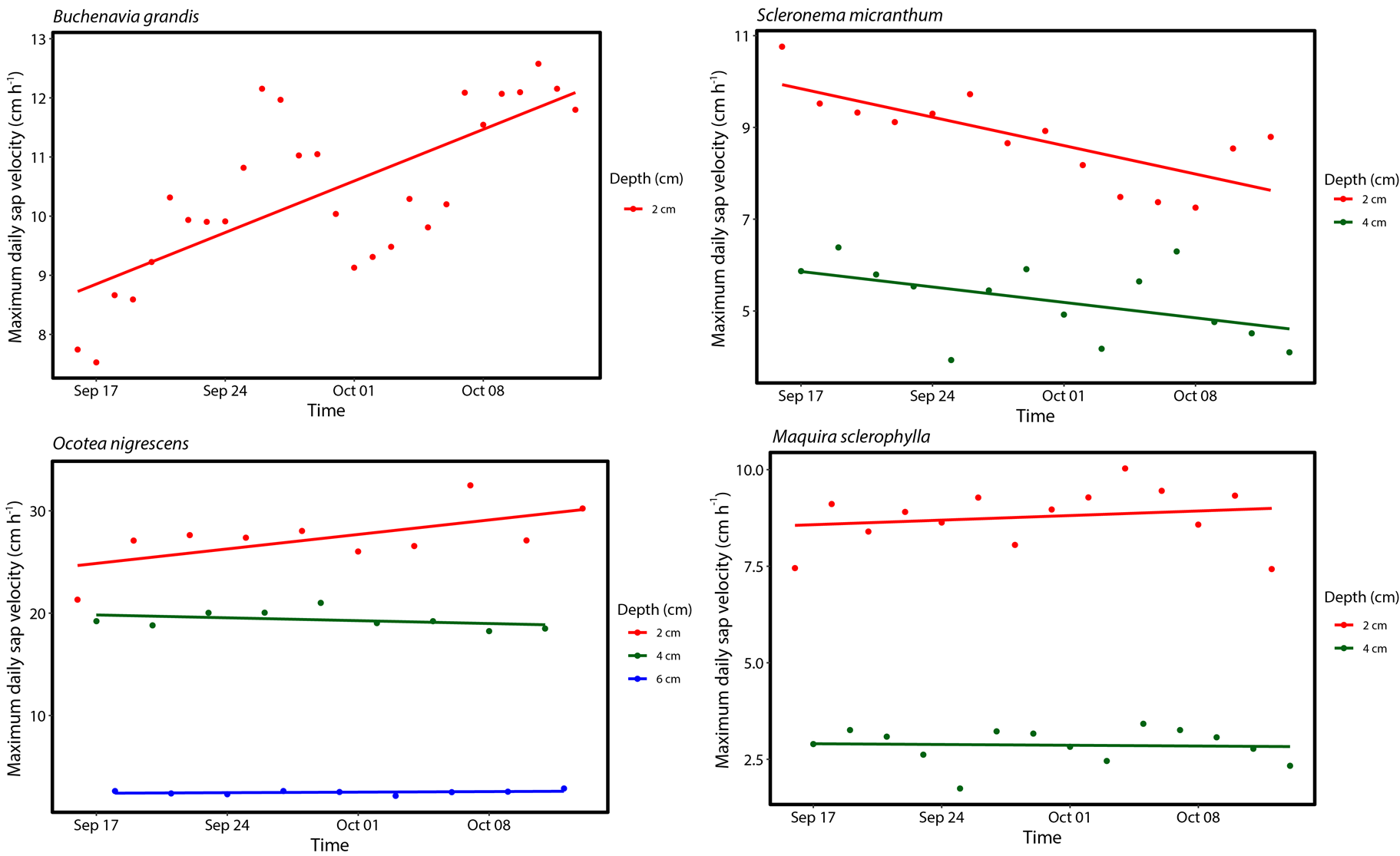


**Figure S3a**. Radial patterns of sap velocity for the largest four sap flow trees during the month-long dry period in 2018.


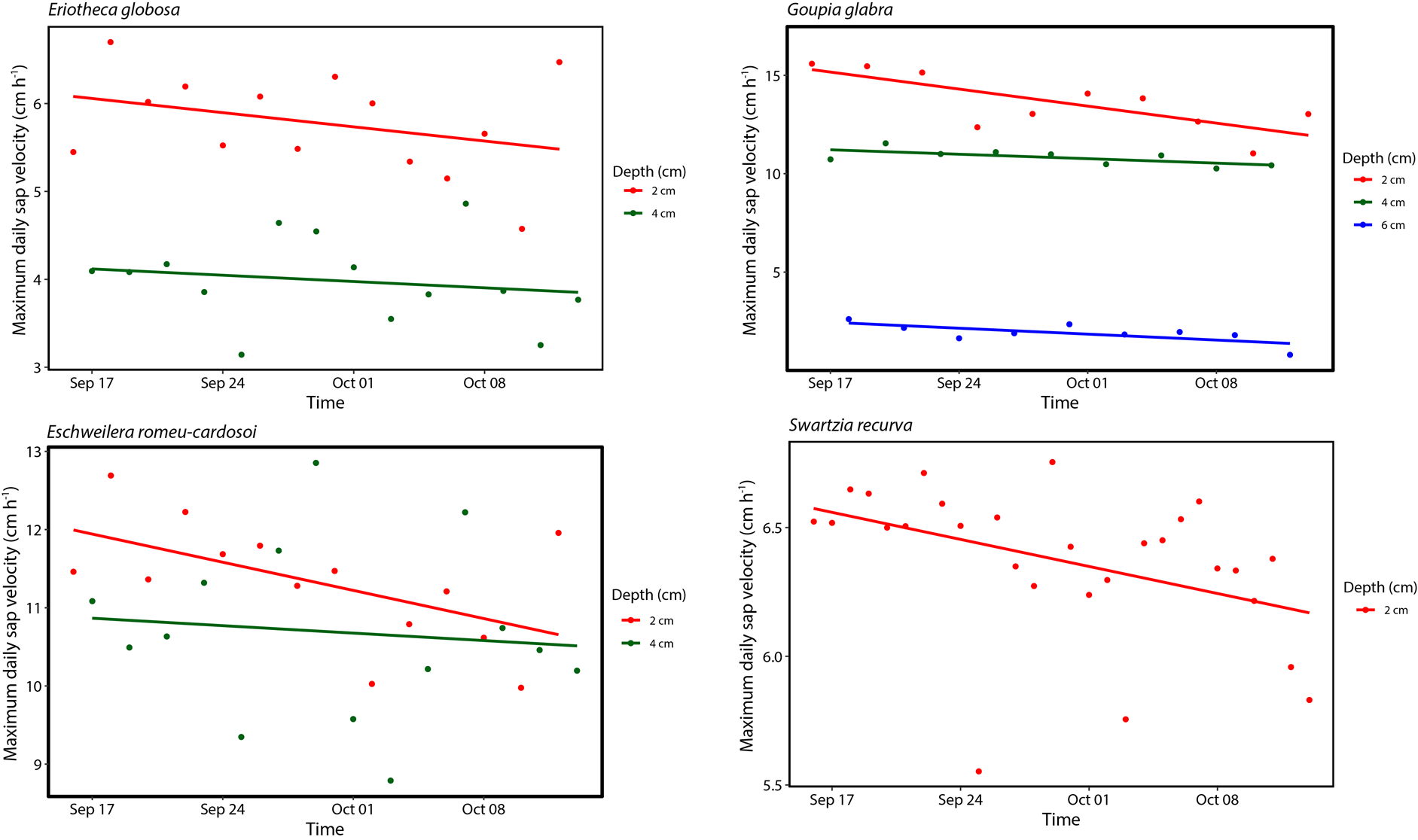
**Figure S3b**. Radial patterns of sap velocity for the smallest four sap flow trees during the month-long dry period in 2018.


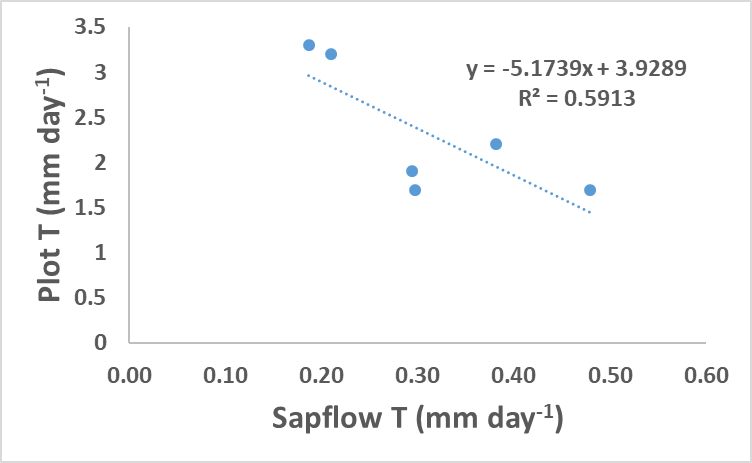


**Figure S4**. Daily plot transpiration based on scaled basal area within a 5 m radius plot (78.5 m^2^) in relation to the single dominant sap flow tree within each plot, scaled based on estimated sap flow tree canopy ground area (range: 37-300 m^2^).
